# Special Issue “Maternal DHA Impact on Child Neurodevelopment”

**DOI:** 10.3390/nu13072209

**Published:** 2021-06-27

**Authors:** Asim K. Duttaroy

**Affiliations:** Department of Nutrition, Institute of Basic Medical Sciences, Faculty of Medicine, University of Oslo, 0317 Oslo, Norway; a.k.duttaroy@medisin.uio.no; Tel.: +47-22-821-547

In this special issue, we have focused on the maternal docosahexaenoic acid, 22:6n-3 (DHA), on children’s neurodevelopment. DHA comprises 10–20% of the total fatty acid composition in the brain, whereas arachidonic acid, 20:4n-6 (ARA), represents 9% [1]. Both DHA and ARA are deposited in large amounts to the fetal brain during the last trimester in utero and continue through the breastfed postnatal life [2]. 

DHA is critically required for the structure and development of the growing fetal brain [1]. Several studies suggest that adequate DHA levels in neural membranes are needed for cortical astrocytes, neurovascular coupling, and metabolism. Many studies have shown that both DHA and ARA and their metabolites are involved in neurogenesis, anti-apoptotic effects, synaptic plasticity, and several signaling and metabolic pathways [2]. In addition to DHA, the importance of ARA in optimal neurodevelopment and growth is well known [2].

The human placental trophoblasts take up maternal plasma free fatty acids (FFAs) via several membranes spanning proteins, such as fatty acid transport protein (FAT) or the cluster of differentiation 36 (CD36), FATPs, plasma membrane fatty acid-binding protein (FABPpm), and intracellular fatty acid-binding proteins (FABPs) [1]. The placenta-specific p-FABPpm is involved in preferential uptake of maternal DHA and ARA for the fetal supply [1,2]. In addition to p-FABPpm, maternal DHA and ARA are also transported by a major facilitator superfamily domain, containing 2a (mfsd2a) of the human placenta but as DHA- [2]. Mfsd2a transports these fatty acids esterified to the lysophosphatidylcholine, but not as FFAs. In addition to these membrane proteins, various cytoplasmic FABPs play important roles at different stages of brain development [2]. However, further work is required to understand the precise roles of these proteins in the transport of DHA and ARA in the feto-placental unit.

Various studies suggest that maternal dietary deficiency of DHA during pregnancy increases the risk for neurocognitive disorders later in the offspring [1]. Most evidence indicates that the DHA accumulation in the brain is mainly influenced by maternal dietary intake, specifically preformed DHA. Insufficient n-3 fatty acids may also lead to DHA deficiency states that could affect the offspring’s metabolic phenotypes by altering placental structure and function, fetal adiposity, body fat distribution, energy utilization, musculoskeletal growth, signaling between brain-adipose tissues, epigenome stability, and inflammation [1]. This underscores the importance of maternal supplementation of DHA during the pregnancy and postnatal period. 

Several randomized trials of maternal DHA supplementation during pregnancy have been carried out. Still, most of the results are inconclusive for several reasons: the background diet of the mothers, lifestyles, and consumption of DHA esterified in different lipid structures such as phospholipids and triglyceride. Several studies compared the bioavailability of triglyceride—or phospholipid-enriched sources of DHA. DHA supplementation in phospholipids or triglycerides during pregnancy can lead to controversial results depending on the models, physiological status, and lipid sources. The intestinal absorption, placental transport, and fetal deposition of DHA may rely on the sources of DHA consumed by the mother. However, there were no consistent differences in fetal accumulation when DHA is provided as phospholipid or triglyceride form. Gazquez and Larque [3] reviewed the DHA bioavailability and its accumulation in both maternal and fetal tissues of supplemented DHA in the form of phospholipids or triglycerides. 

Khandelwal et al. [4] reported the impact of maternal supplementation of 400 mg algal DHA/day on newborn anthropometry; appearance, pulse, grimace, activity, and respiration (APGAR) score; duration of gestation; and birth weight among Indian women. They supplemented women (*n* = 478) from 14–20 weeks’ gestation till delivery in a double-blind, randomized, placebo-controlled (placebo; *n* = 479) trial in pregnant women. They did not find any significant differences between DHA and placebo groups for birth weight, length, or head circumference. The mean gestational age at delivery and APGAR scores were similar between the two groups. They concluded that supplementing pregnant mothers with 400 mg/day DHA did not impact the offspring’s birth weight, length, or head circumference [4]. 

In a continuation of the above study, Khandelwal et al. [5] investigated the effects of maternal DHA supplementation during pregnancy and lactation on offspring’s neurodevelopment. They examined the effects of 400 mg algal DHA/day maternal supplementation on offspring’s neurodevelopment at 12 months in a double-blind, randomized, placebo-controlled trial involving pregnant Indian women from ≤20 weeks of gestation through 6 months postpartum. The primary outcome was infant neurodevelopment at 12 months, assessed using the Development Assessment Scale for Indian Infants (DASII). At 12 months, the mean development quotient scores in the DHA and placebo groups were not statistically significant. This study also concluded that supplementing Indian mothers through pregnancy and lactation with 400 mg/d DHA did not impact offspring neurodevelopment at 12 months of age compared with the placebo group. 

## Data Availability

Not applicable.
